# The complete mitochondrial genome and phylogenetic analysis of *Phintella cavaleriei* (Araneae: Salticidae)

**DOI:** 10.1080/23802359.2021.1927224

**Published:** 2021-05-12

**Authors:** Peng-Liang Xia, Yong Huang, Xiao-Fei Xie, Shu-Yan Yan, Yi Luo, Wen-Jia Yang

**Affiliations:** a Enshi Tobacco Company of Hubei Province, Enshi, China; b Guizhou Provincial Key Laboratory for Rare Animal and Economic Insect of the Mountainous Region, College of Biology and Environmental Engineering, Guiyang University, Guiyang, China

**Keywords:** *Phintella cavaleriei*, Salticidae, mitogenome

## Abstract

The complete mitochondrial genome of *Phintella cavaleriei* is 14,325 bp in length, containing 13 protein-coding genes (PCGs), 22 transfer RNA genes (tRNAs), two ribosomal RNA genes, and a putative control region. The overall nucleotide composition is 35.04% A, 8.46% C, 13.41% G, and 43.09% T, with a total of A + T content of 78.13%. Ten PCGs start with typical ATN codons, two genes (*cox2* and *cox3*) begin with TTG, and *cox1* use TTA as initiation codon. Ten PCGs use usual termination codon of TAA or TAG, whereas the remaining three PCGs had an incomplete termination codon (T—). Seven tRNAs (*trnY*, *trnC*, *trnG*, *trnN*, *trnH*, *trnP*, and *trnV*) lacked the TΨC arm stem, while two tRNAs (*trnS_1_* and *trnS_2_*) lost the dihydrouracil (DHU) arm. Phylogenetic analysis based on 13 PCGs indicated that *P. cavaleriei* was closely related to *Cheliceroides longipalpis*, and clustered within Salticidae clade.

The jumping spider *Phintella cavaleriei* belongs to the family of Salticidae, which includes 6329 described species in 659 genera (Wheeler et al. 2017; Kanesharatnam and Benjamin 2019; World Spider Catalog 2021). *Phintella cavaleriei* is an important predator of many agricultural pests and mainly distributed in China and Korea (Cui et al. 2012; Kim and Lee 2014). In this study, adult individuals of *P. cavaleriei* were collected from organic tobacco fields in Enshi City, Hubei Province, China (N30°20i, E109°25E). Samples were preserved in 95% ethanol and stored in the Natural Enemy Insect Specimen Room of Enshi Tobacco Company under the voucher number ETC-2020-01. Genomic DNA was extracted from whole body of a single specimen using the EasyPure^®^ Genomic DNA Kit (TransGen, Beijing, China). The complete mitogenome sequence of *P. cavaleriei* was amplified by polymerase chain reaction (PCR) with the LA PCR™ Kit (TaKaRa Bio Inc, Dalian, China). Initially, partial sequences of *cox1*, *cox3*, *nd1*, *nd5*, *12S rRNA*, and *16S rRNA* were amplified by using the universal primers, which were designed on the basis of the conserved regions of 10 arachnida mitogenomes. These fragments were then used for designing six specifc primers to amplify the remaining mitogenomic sequences in several PCR steps.

The complete mitogenome of *P. cavaleriei* (GenBank accession number MW540530) is a typical circular DNA molecule of 14,325 bp in length, and contains 13 protein-coding genes (PCGs), 22 transfer RNA genes (tRNAs), two ribosomal RNA genes (*12S rRNA* and *16S rRNA*), and a putative control region (Boore 1999). The gene order and orientation of *P. cavaleriei* are identical with other spider mitogenomes (Pan et al. 2016; Wang et al. 2016). The overall base composition of *P. cavaleriei* mitogenome is A (35.04%), C (8.46%), G (13.41%), and T (43.09%), with a total of A + T content of 78.13%. The AT-skew and GC-skew of this genome were −0.103 and 0.226, respectively. Gene overlaps were found in 22 locations and their total length was 302 bp. The longest overlap was 35 bp in length and resided between *nad4* and *nad4L.* There were 6 intergenic spacer regions comprising a total of 122 bp and the largest spacer (70 bp) resided between *trnA* and *trnN*. The length of 22 tRNAs ranged from 49 bp (*trnC*) to 80 bp (*trnM*), A + T content ranged from 69.81% (*trnN*) to 87.93% (*trnT*). Nine tRNAs lacked the potential to form the cloverleaf-shaped secondary structure. Seven of them (*trnY*, *trnC*, *trnG*, *trnN*, *trnH*, *trnP*, and *trnV*) lacked the TΨC arm stem, two tRNAs (*trnS_1_* and *trnS_2_*) lost the dihydrouracil (DHU) arm. The *16S rRNA* (1036 bp) was located between *trnL_1_* and *trnV*, and *12S rRNA* (692 bp) resided between *trnV* and *trnQ,* and their A + T contents were 83.01% and 81.36%, respectively. The control region was located between *trnQ* and *trnM* genes with a length of 663 bp, and the A + T content was 79.03%.

Among the 13 PCGs, *nad1*, *nad4*, *nad4L*, and *nad5* were encoded on the light strand (L-strand), while the remaining nine genes were encoded on the heavy strand (H-strand). The A + T content of these 13 PCGs ranged from 72.01% (*cox1*) to 88.24% (*atp8*). The *cox1* initiated with TTA as the start codon, *cox2* and *cox3* started with TTG, *atp8*, *nad2*, *nad4L*, *nad5*, and *nad6* started with ATT, and the remaining five PCGs (*atp6*, *cob*, *nad1, nad4*, and *nad6*) started with ATA. Ten PCGs terminated with conventional stop codons (TAA and TAG), while *nad2*, *nad4*, and *cob* used incomplete codon (T—) as termination codon. Based on the concatenated amino acid sequences of 13 PCGs, the neighbor-joining method was used to construct the phylogenetic relationship of *P. cavaleriei* with 20 other spiders by MEGA7 (Kumar et al. 2016). The result showed that *P. cavaleriei* was closely related to *Cheliceroides longipalpis*, and clustered within Salticidae clade (Figure 1).

**Figure 1.**
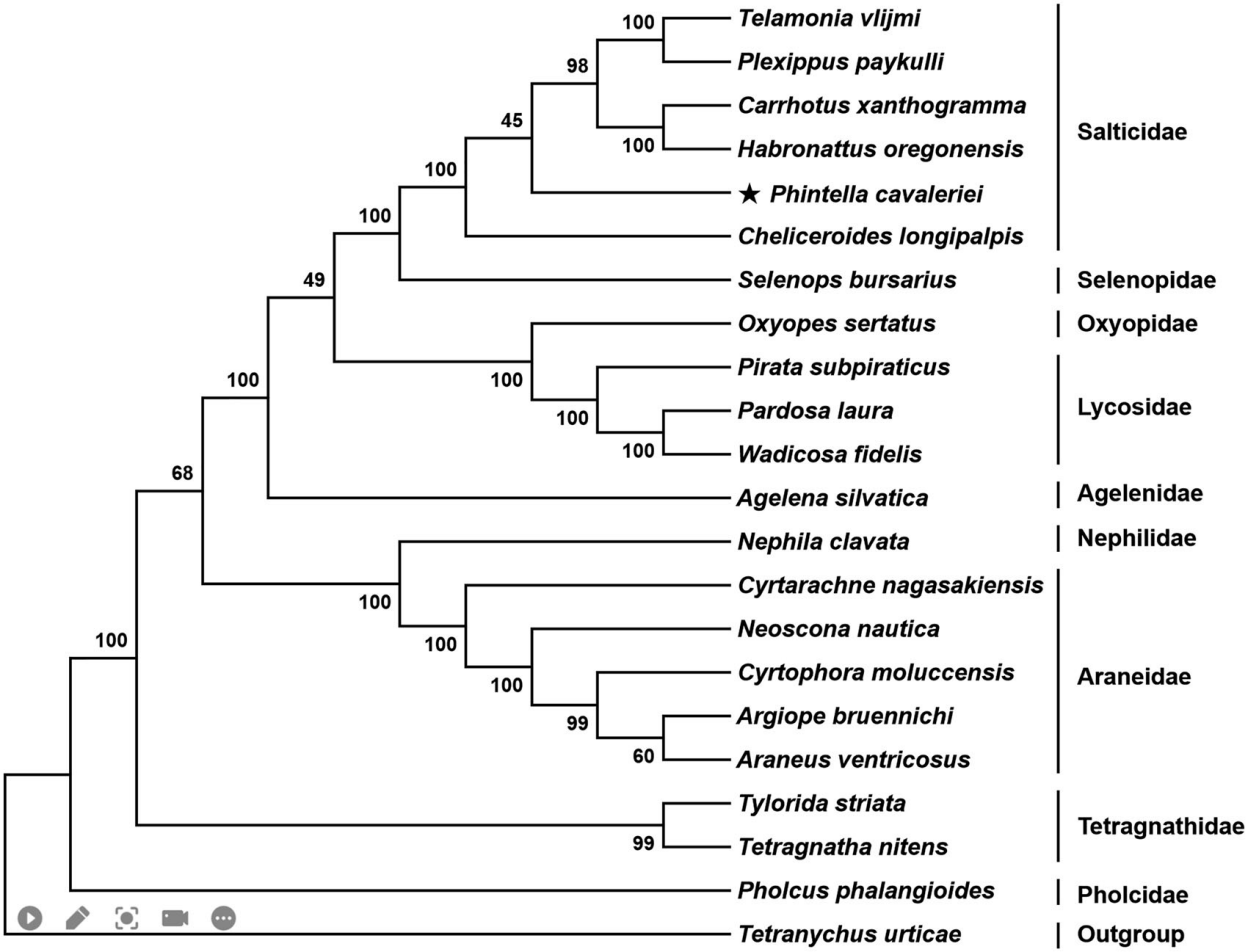
Phylogenetic tree showing the relationship between *Phintella cavaleriei* and 20 other representative spiders based on neighbor-joining method. GenBank accession numbers used in the study are the following: *Agelena silvatica* (KX290739), *Araneus ventricosus* (NC_025634), *Argiope bruennichi* (NC_024281), *Carrhotus xanthogramma* (NC_005942), *Cheliceroides longipalpis* (MH891570), *Cyrtarachne nagasakiensis* (KR259802), *Cyrtophora moluccensis* (KM820884), *Habronattus oregonensis* (AY571145), *Neoscona nautica* (KR259804), *Nephila clavata* (NC_008063), *Oxyopes sertatus* (KM272950), *Pardosa laura* (KM272948), *Phintella cavaleriei* (MW540530), *Pholcus phalangioides* (NC_020324), *Pirata subpiraticus* (NC_025523), *Plexippus paykulli* (NC_024877), *Selenops bursarius* (NC_024878), *Telamonia vlijmi* (NC_024287), *Tetragnatha nitens* (KP306790), *Tetrancychus urticae* (EU345430.1), *Tylorida striata* (MN615900), and *Wadicosa fidelis* (NC_026123). *T. urticae* was used as an outgroup. Spiders determined in this study was marked with an asterisk.

## Data Availability

The data that support the findings of this study are openly available in GenBank of NCBI at https://www.ncbi.nlm.nih.gov under the accession number MW540530.
